# “DO IT ANYWAY”—RESIDENT-DRIVEN ENGAGEMENT TO MAINTAIN MEANINGFUL LIFE IN CONTINUING-CARE RETIREMENT COMMUNITIES

**DOI:** 10.1093/geroni/igad104.1158

**Published:** 2023-12-21

**Authors:** Xiaoli Li

**Affiliations:** Southern Illinois University, Carbondale, Illinois, United States

## Abstract

As the aging population continues to grow, the demand for Continuing Care Retirement Communities (CCRCs) is increasing. However, residents in CCRCs often face social isolation and loneliness, which can negatively impact their health and well-being. To combat these issues, activities and engagement have been proposed as potential solutions. However, the voices of those vulnerable to social isolation are seldom heard. This study aimed to explore the perspective of residents on how resident-driven engagement in CCRCs may maintain a meaningful life. A qualitative research approach was used, consisting of semi-structured interviews with 26 residents in CCRC. Thematic analysis identified five key themes related to resident-driven engagement: a sense of community, turning lemons into lemonade, doing it anyway, pink letters, and committees and clubs. The “do it anyway” attitude emerged as a prevalent theme, emphasizing the importance of pushing past challenges and limitations to pursue activities that bring joy and meaning to residents’ lives. The findings suggest that resident-driven engagement can lead to a sense of purpose, belonging, and fulfillment in CCRCs. The research provides insights into how communities can foster an environment that supports such engagement. By prioritizing resident-driven activities and empowering residents to take control of their lives, CCRCs can promote the well-being and quality of life of their residents.

